# Optimization of biochar preparation from the stem of *Eichhornia crassipes* using response surface methodology on adsorption of Cd^2+^

**DOI:** 10.1038/s41598-019-54105-1

**Published:** 2019-11-26

**Authors:** Runjuan Zhou, Ming Zhang, Jinhong Zhou, Jinpeng Wang

**Affiliations:** College of Electrical Engineering, Anhui Polytechnic University, Wuhu, 241000 Anhui P.R. China

**Keywords:** Pollution remediation, Characterization and analytical techniques

## Abstract

In this study, preparation of *Eichhornia crassipes* stem biochar (ECSBC) was optimized and applied for the removal of Cd^2+^ from aqueous solution. To obtain the best adsorption capacity of ECSBC, the response surface methodology (RSM) was used to optimize the preparation conditions of ECSBC (OECSBC). The interactions among heating time (*X*_1_), heating temperature (*X*_2_) and heating rate (*X*_3_) were designed by Box-Behnken Design (BBD) experiments. The software gave seventeen runs experiment within the optimal conditions towards two response variables (removal rate and adsorption capacity for Cd^2+^). The results showed that the mathematical model could fit the experimental data very well and the significance of the influence factors followed the order as heating temperature (*X*_2_) > heating rate (*X*_3_) > heating time (*X*_1_), and the influence of interaction term is: *X*_1_ and *X*_2_ (heating time and heating temperature) > *X*_2_ and *X*_3_ (heating temperature and heating rate) > *X*_1_ and *X*_3_ (heating time and heating rate). Based on the analysis of variance and the method of numerical expected function, the optimal conditions were heating time of 2.42 h, heating temperature of 393 °C, and heating rate of 15.56 °C/min. Under the optimum conditions, the predicted the maximum removal rate and adsorption capacity were 85.2724% and 21.168 mg/g, respectively, and the experimental value of removal rate and adsorption capacity for Cd^2+^ were 80.70% and 20.175 mg/g, respectively, the deviation from the predicted value were 5.36% and 4.69%. The results confirmed that the RSM can optimize the preparation conditions of ECSBC, and the adsorption capacity of OECSB was improved.

## Introduction

Biochar (BC), a carbon-rich solid which is defined as “a solid material obtained from the thermochemical conversion of biomass in an oxygen-limited environment” and produced from various feedstocks, such as wood biomass, animal manure, crop residues, and solid wastes^1,2^. At present, biochar has been applied in pollutant removal, carbon sequestration and soil improvement, and has attracted extensive attention^3^. In these applications, biochar has the properties of large specific surface area, porous structure, enriched surface functional groups and mineral composition, and it can be used as a suitable adsorbent for the removal of pollutants in aqueous solutions^4^.

BC is usually produced through a variety of thermochemical methods, including slow pyrolysis, fast pyrolysis, hydrothermal carbonization, torrefaction and gasification^5–7^. Among these methods, pyrolysis is a usually method in production, and it is a process for decomposing organic materials thermally under limited-oxygen conditions in the temperature range 300–900 °C^8^. The pyrolysis conditions (e.g. heating temperature, heating time, and heating rate) have great influence on the properties of biochar. Many scholars have done a lot of research on the components and structural characteristics of biochar under various pyrolysis conditions. These results show that heating temperature, heating time and heating rate are the three most important factors in the preparation of biochar^9–12^. For instance, the yield, surface area, pore volume, elemental composition, and calorific value of BC are varied with heating temperature^13^. Studies have been reported that the functional groups and the yield of BC decreased with increasing heating temperature^14–17^. The residence time has effected on the product composition, surface areas, and pore characteristics of BC^17^, and the heating rate mainly effect on yields, the fixed and operation cost of BC^13^. Therefore, there are many studies on the heating temperature, heating time and heating rate in the pyrolysis process of BC^15,16,18–20^.

However, the present studies were mainly on the respective effects of three factors on the properties of biochar and not consider the interaction of three factors. Therefore, it is necessary to study the interaction between the preparation conditions of biochar to improve its adsorption performance. In general, the full factorial experiments can solve the problem of multiple variables among the process, but it’s a time-consuming and expensive process^21^. In order to obtain the interaction of the three factors during the preparation of BC, the response surface methodology (RSM) is used for optimizing and improving products for the actual production process. The RSM is useful for solving the multiple variables problems that affect the processing indicators (e.g. surface area, adsorption capacity)^22,23^. Through the design of experiments, the RSM reduce the number of experimental runs, and establish the regression model and improve the model for operating conditions^22,24,25^. For the three-level factorial designs, the RSM have a set of mathematical and statistical techniques, included Central Composite (CCD), Box-Behnken Design (BBD) and Doehlert Matrix^25^. In recent years, utilization of the RSM has been increased for the optimization of the preparation of adsorbents. For example, adsorbent from rice bran^26^, agricultural wastes-derived biochars^27^, activated carbon was prepared by cassava stem^19^, were optimized using RSM based on the experiment designs.

Cd^2+^ is a highly toxic heavy metal which is reported carcinogen substance to human beings by International Agency for Research on Cancer (IARC, 1974)^28^. Cd^2+^ is very harmful to kidney, prostate, pancrease, lungs, bones, and the famous itai-itai disease is caused by Cd^2+^^29^. Nowadays, adsorption is mostly used method for removal of heavy metals, so Cd^2+^ can also be treated by adsorption. *Eichhornia crassipes* is a kind of perennial aquatic plant which grows rapidly and floats freely, and it has high tolerance and absorption ability to heavy metals^30^. To our knowledge, although lot of studies have used *Eichhornia crassipes* as adsorbent for heavy metal removal^31–34^, while no study has been reported on preparation conditions of biochar which from *Eichhornia crassipes* stem for the adsorption of heavy metals from aqueous solutions by using an optimization method, for instance Box-Behnken design (BBD), a subset of response surface methodology.

In this study, the RSM was used to optimize the preparation conditions of *Eichhornia crassipes* stem biochar (ECSBC). The main objectives of this study are to obtain the optimum preparation conditions (heating time, heating temperature, and heating rate) for the production process of ECSBC. In order to explore the interaction between preparation conditions, a Box-Behnken design under the RSM was employed to find the optimum preparation conditions of production process. The quadratic and cubic polynomial regression models are used to involve three independent factors (heating time, heating temperature, and heating rate), and the removal rate and adsorption capacity of Cd^2+^ as the main response. Based on optimal conditions, the experimental value of the removal rate and adsorption capacity of Cd^2+^ by the prepared ECSBC were very close to the predicted value of the model. Then, physical characteristics of the samples were characterized. These results showed that the response surface methodology is suitable for the optimization of conditions in the preparation process of biochar.

## Materials and Methods

### Materials

Plant samples of *Eichhornia crassipes* in this study were obtained from the pond in Anhui Polytechnic University in Wuhu, Anhui city of China. A simulated solution containing 1000 mg/L of Cd^2+^ was prepared by dissolving 2.0316 g of CdCl_2_·2.5H_2_O in 1000 ml ultrapure water and the ultrapure water was used through all experiments in this work. All working solutions in this study were obtained by dilution of the simulated solution, and the pH values of all working solutions were adjusted using 0.1 mol/L NaOH or 0.1 mol/L HCl solution. All chemical agents were of analytical grade and purchased from Sinopharm Chemical Reagent Co., Ltd., Shanghai, China.

### Preparation of ECSBC

The pre-treatment process of raw materials of *Eichhornia crassipes* were firstly washed by tap water, and secondly cleaned with ultrapure water, and then used scissors to separate the stems, leaves and roots of *Eichhornia crassipes*. Subsequently, the selected stems were crushed to smaller sizes in the range of 1–3 mm, and dried in drying oven at 105 °C for 24 h. A crucible cup in a muffle furnace was used for production of the ECSBC. The heating time, heating temperature and heating rate could be controlled by the muffle furnace. During the production process, the crucible cup with a lid was wrapped with tin foil to isolate the oxygen. The achieved ECSBC was kept in airtight bottle for subsequent experiments.

### Single-factor experiments

A series of single-factor experiments were conducted to study the effect of three factors on adsorptive property of ECSBC. For this purpose, the removal rate and adsorption capacity of Cd^2+^ on heating time, heating temperature and heating rate were investigated.

### Response surface methodology (RSM): The box-behnken experimental design

In order to avoid the traditional experiments (one factor at a time) along with optimizing a process through the individual and interactive effects of independent variables simultaneously, the optimize design of experiments can be used to solve this problem^35,36^. RSM is a multivariate statistical technique for optimizing process variables and their responses^37^. One of the widely used RSM methods is the Box-Behnken Design (BBD)^38–40^. In this study, RSM was used to optimize the preparation conditions of ECSBC. The Box-Behnken design was employed to optimize the ECSBC preparation parameters such as heating time, heating temperature and heating rate. For each parameter, the central values were obtained from single-factor experiments. In the design, 17 samples of ECSBC were prepared and tested for the adsorption of 50 mg/L of Cd^2+^ solutions. In the RSM method, to predict the optimum conditions for the preparation of ECSBC and to express the interaction between dependent and independent factors, the mathematical quadratic model shown in Eq. (1)^41^:

1 $$Y={\beta }_{0}+\mathop{\sum }\limits_{j=1}^{3}{\beta }_{j}\,{X}_{j}+\mathop{\sum }\limits_{j=1}^{3}{\beta }_{jj}\,{X}_{j}^{2}+\mathop{\sum }\limits_{i=1}^{3}\mathop{\sum }\limits_{j=1}^{3}{\beta }_{ij}\,{X}_{i}\,{X}_{j}+\varepsilon $$

where *Y* is the predicted response; *β*_0_ is the constant; *β*_*j*_, *β*_*jj*_, and *β*_*ij*_ refer to coefficients of linear effect, quadratic effect and interaction effect, respectively; *ε* is a random error; *X*_*i*_ and *X*_*j*_ are dimensionless coded predicted values for the independent factors. Analysis of variance (ANOVA) is used to forecast the applicability of quadratic model and the significance of each item in the equation. The Design-Expert 10.0 was applied for experimental design and data analysis. The ECSBC was prepared in the optimization of conditions which was called OECSBC.

### Adsorption studies

The adsorption performance of all prepared ECSBC was tested in batch experiments. The batch experiments were performed in 250 ml conical flask containing 100 ml of Cd^2+^ shaken at 150 rpm/min for 3 h at 298 K with 0.2 g ECSBC, and the initial concentration of Cd^2+^ in all single-factor experiments solutions were 20 mg/L, and in BBD experimental design, the initial concentration of Cd^2+^ were 50 mg/L. 0.1 mol/L solutions of HCl and NaOH were used to adjust the pH of solutions. After the adsorption experiments, samples were immediately filtered through a syringe filter (0.45 μm), and residual concentrations of Cd^2+^ in solution were measured by Shimadzu AA-7000G atomic absorption spectrophotometer (Shimadzu, Japan)^42^.

The removal rate (*R%*) of Cd^2+^ and the adsorption capacity (*q*_*t*_) were determined using the following Eqs. (2) and (3) ^43^, respectively:

2 $$R=\frac{{C}_{0}-{C}_{t}}{{C}_{0}}\times 100$$

3 $${q}_{t}=\frac{({C}_{0}-{C}_{t})V}{m}$$

*C*_0_ and *C*_*t*_ are the concentrations of Cd^2+^ at initial and *t* time (mg/L), respectively; *m* is the mass of ECSBC (g).

### Characterization of OECSBC

The Zeta potential of OECSBC was determined by Zeta potentiometer (Zetasizer Nano ZEN3690, Malvern, England), and the pH of OECSBC was determined by pH meter (PHS-25, LEICI, China). The Surface morphology and element composition of OECSBC was imaged using Hitachi S-4800 scanning electron microscopy (SEM) with energy dispersive X-Ray (EDX) spectroscopy (Hitachi, Japan). Specific surface area was determined using a surface area and porosity analyzer (Quantachrome, United States). The IRPrestige-21 transform infrared spectrometer (Shimadzu, Japan) was used to analyze the functional groups on three adsorbents surfaces. The composition and structure of the atoms or molecules inside OECSBC were characterized by XRD with Bruck-D8 series X-ray (powder) diffractometer (Bruck, German).

### Analysis methods

The data and figures of preliminary experiments were analyzed by OriginPro 2017. The optimize experiments data and figures were designed by the soft of Design-Expert 10.0.

## Results and Discussion

### Single-factor experiments results

#### Effects of heating time on adsorption property of ECSBC

**Figure 1:**
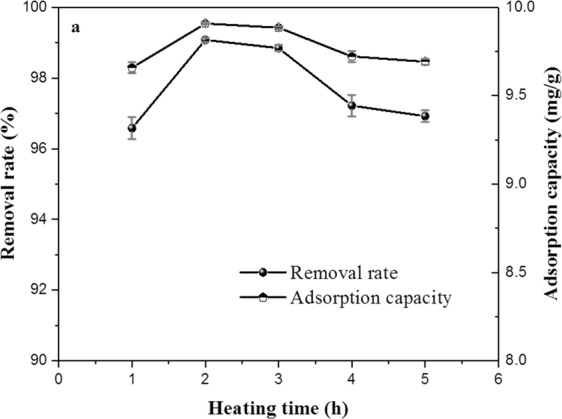
Effect of heating time on the adsorption property of ECSBC.

To explore the effect of heating time on property of ECSBC, experiments were carried out in the heating time range from 1 h-5 h at heating temperature of 400 °C, heating rate of 15 °C/min. The results are displayed in Fig. 1.

Figure 1 showed the removal rate and adsorption capacity of Cd^2+^ by ECSBC increased from 96.23% to 99.045% and 9.63 mg/g to 9.9045 mg/g with the increase in heating time from 1 to 2 h, respectively. As the heating time increased, the adsorption performance of ECSBC was reduced. When the heating time increased to 5 h, the removal rate and adsorption capacity of Cd^2+^ were 96.8% and 9.68 mg/g, respectively. Therefore, 2 h heating time was selected as the center value in the optimization experiment.

#### Effect of heating temperature on adsorption property of ECSBC

**Figure 2:**
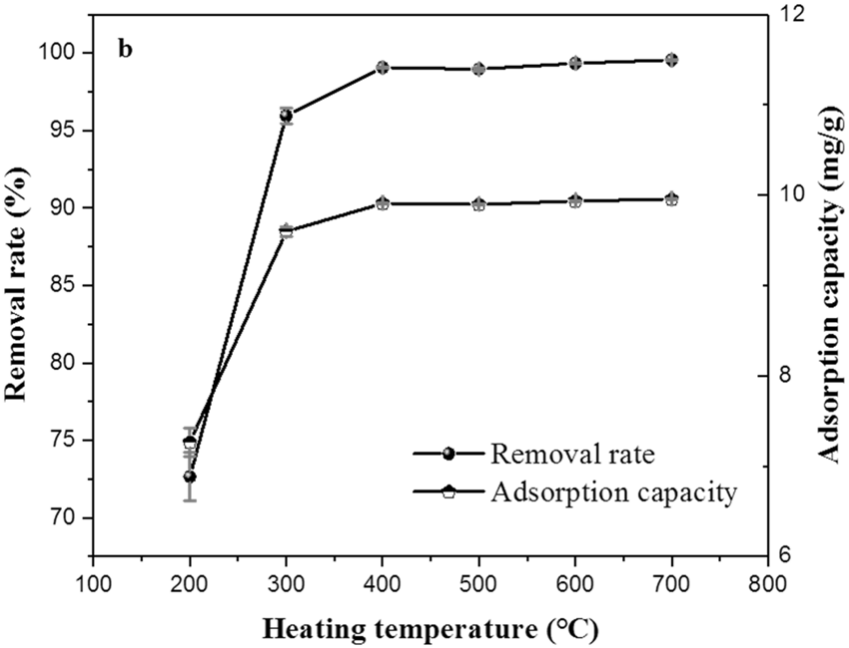
Effect of heating temperature on the adsorption property of ECSBC.

The experiments of heating temperature were performed at heating temperature range from 200–700 °C, and other conditions kept constant, heating time 2 h, heating rate 15 °C/min. The results are showed in Fig. 2.

Figure 2 displayed the effects of heating temperature on adsorption performance of ECSBC. When the heating temperature increased from 200 to 400 °C, the removal rate and adsorption of Cd^2+^ increased from 72.65% and 7.265 mg/g to 99.07% and 9.907 mg/g, respectively. However, when the heating temperature increased to 500 °C, the removal rate and adsorption capacity decreased to 98.97% and 9.897 mg/g. With increasing the heating temperature to 600 °C and 700 °C, compared with the removal rate and adsorption capacity of 400 °C, the removal rate and adsorption capacity were increased. Although the adsorption effect increased, the increase was not significant, and combined with energy consumption, so the 400 °C was chosen for the center value of heating temperature for the optimization design experiments.

### Effect of heating rate on adsorption property of ECSBC

**Figure 3:**
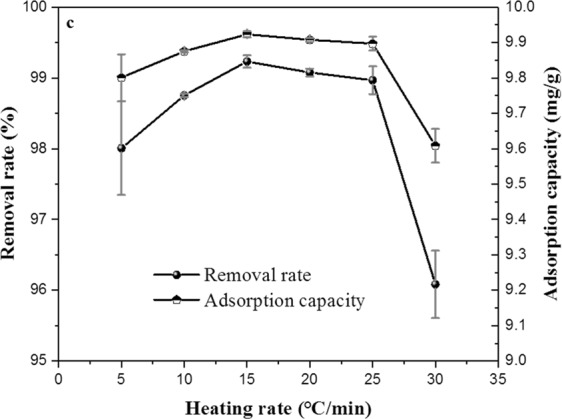
Effect of heating rate on the adsorption property of ECSBC.

The heating rate as the preparation condition of biochar has been rarely studied. In this study, the effect of heating rate on adsorption property of ECSBC was studied, the rate were ranged from 5–30 °C/min, kept heating time 2 h, heating temperature 400 °C. The results are presented in Fig. 3.

From Fig. 3, under the condition of 15 °C/min, the removal rate and adsorption capacity of Cd^2+^were 99.23% and 9.923 mg/g, respectively. Results are highest in all heating rate. This heating rate belong to slow pyrolysis (<10 °C/s)^44^, and this results consistent with the study of Tan *et al*.^4^. Therefore, the heating rate of 15 °C/min was chosen as a center value of the optimization design.

### Box-Behnken design (BBD) and model analysis

According to the center values of the single-factor experiments, BBD was applied to optimize experiments, and the experimental design of three variables and three levels is shown in Table 1.

**Table 1:** Coded and actual levels for indpendent variable and levels.

Variables	Symbol	Coded levels		
		−1	0	1
Heating time (h)	*X*_1_	1	2	3
Heating temperature( °C)	*X*_2_	300	400	500
Heating rate (°C/min)	*X*_3_	10	15	20

In order to statistically optimize the preparation conditions (heating time, heating temperature, and heating rate) and estimate the effects of these factors on removal rate and adsorption capacity of Cd^2+^ by ECSBC, seventeen experimental runs were conducted and the experimental results are arranged in Table 2. The resulting of quadratic regression model equations for removal rate (*Y*_*R%*_) and adsorption capacity (*Y*_*q*_) are given as Eq. (4) and Eq. (5), respectively.

4 $$\begin{array}{ccc}{Y}_{R \% } & = & 84.42+1.19{X}_{1}-1.03{X}_{2}+1.54{X}_{3}-2.20{X}_{1}{X}_{2}+1.47{X}_{1}{X}_{3}\\  &  & -2.06{X}_{2}{X}_{3}-2.33{X}_{1}^{2}-21.78{X}_{2}^{2}-9.63{X}_{3}^{2}\end{array}$$

5 $$\begin{array}{c}{Y}_{q}=21.10+0.30{X}_{1}-0.26{X}_{2}+0.38{X}_{3}-0.55{X}_{1}{X}_{2}+0.37{X}_{1}{X}_{3}\\ \,\,\,-0.51{X}_{2}{X}_{3}-0.58{X}_{1}^{2}-5.44{X}_{2}^{2}-2.41{X}_{3}^{2}\end{array}$$

**Table 2:** Box-Behnken experiment design and results with independent variables.

Run	*X*_1_	*X*_2_	*X*_3_	Adsorption capacity(mg/g)	Removal rate (%)
1	−1	−1	0	14.08	56.30
2	1	1	0	14.98	59.92
3	0	0	0	21.19	84.77
4	−1	1	0	16.09	64.35
5	0	1	1	12.03	48.13
6	0	0	0	20.93	83.73
7	0	0	0	21.09	84.37
8	0	0	0	20.95	83.81
9	0	0	0	21.35	85.41
10	−1	0	1	17.65	70.60
11	1	0	1	19.58	78.33
12	1	0	−1	17.84	71.37
13	−1	0	−1	17.38	69.52
14	1	−1	0	15.17	60.67
15	0	1	−1	12.53	50.12
16	0	−1	−1	13.44	53.77
17	0	−1	1	15.01	60.02

The analysis of variance (ANOVA) by quadratic regression model which for removal rate and adsorption capacity are listed in Table 3. The Prob > *F* values < 0.05 indicate the significance of model terms^45^, the model *F* value of removal rate and adsorption capacity was 25.51, while Prob > *F* = 0.0002 < 0.05, indicating that the model was significantly established. The correlation coefficient *R*^2^ of the two models were 0.9704 > 0.8, indicating that the model fitted the experimental data well and the experimental error was small. In addition, *R*^2^_*adj*_ = 0.9324, indicating that the model can explain the change of response value of 93.24%. The SNR of the model was 13.060 > 4, which also indicates that the model provides a reliable signal to respond to the experimental design of ECSBC preparation for Cd^2+^ adsorption. As can be seen from Table 3, *X*_2_^2^ and *X*_3_^2^ are significant terms, while *X*_1_, *X*_2_, *X*_3_, *X*_1_*X*_2_, *X*_1_*X*_3_, *X*_2_*X*_3_ and *X*_1_^2^ were all non-significant terms, indicating that the quadratic regression model is not very good at fitting experimental data.

**Table 3:** The quadratic regression model of ANOVA for removal rate and adsorption capacity of Cd^2+^ by ECSBC.

Sources	Sum of Squares	df	Mean Square	F-value	p-value Prob. >F
(a) Remvoval rate Model	2641.63	9	293.51	25.51	0.0002
*X*_1_	11.32	1	11.32	0.98	0.3543
*X*_2_	8.47	1	8.47	0.74	0.4193
*X*_3_	18.96	1	18.96	1.65	0.2401
*X*_1_*X*_2_	19.36	1	19.36	1.68	0.2357
*X*_1_*X*_3_	8.65	1	8.65	0.75	0.4146
*X*_2_*X*_3_	16.95	1	16.95	1.47	0.2643
*X*_1_^2^	22.90	1	22.90	1.99	0.2012
*X*_2_^2^	1996.99	1	1996.99	173.53	<0.0001
*X*_3_^2^	390.65	1	390.65	33.95	0.0006
Residual	80.56	7	11.51	—	—
Lack of fit	78.60	3	26.20	53.56	0.0011
Pure error	1.96	4	0.49	—	—
Cor total	2722.19	16	—	—	—
**(b) Adsorption capactiy**					
Model	165.10	9	18.34	25.51	0.0002
*X*_1_	0.71	1	0.71	0.98	0.3543
*X*_2_	0.53	1	0.53	0.74	0.4193
*X*_3_	1.19	1	1.19	1.65	0.2401
*X*_1_*X*_2_	1.21	1	1.21	1.68	0.2357
*X*_1_*X*_3_	0.54	1	0.54	0.75	0.4146
*X*_2_*X*_3_	1.06	1	1.06	1.47	0.2643
*X*_1_^2^	1.43	1	1.43	1.99	0.2012
*X*_2_^2^	124.81	1	124.81	173.53	< 0.0001
*X*_3_^2^	24.42	1	24.42	33.95	0.0006
Residual	5.03	7	0.72	—	—
Lack of fit	4.91	3	1.64	53.56	0.0011
Pure error	0.12	4	0.031	—	—
Cor total	170.14	16	—	—	—

Generally speaking, when the quadratic regression model is not well fitted, the cubic regression model can be used. The regression equations fitted by the cubic regression model are shown in Eq. (6) and Eq. (7). The ANOVA by cubic regression model which for removal rate and adsorption capacity are listed in Table 4. From Table 4, the *F* value of the cubic regression model is 463.39 and Prop > *F* < 0.0001 means that the model is more significant than the quadratic regression model. The correlation coefficient of model fitting *R*^2^ = 0.9993, *R*^2^_*adj*_ = 0.9971, and the SNR of the model 59.326 is much higher than 4, and these above data indicated that the cubic regression model could be well fit the experimental results. In the three regression models, the *F* values of heating time (*X*_1_), heating temperature (*X*_2_) and heating rate (*X*_3_) are 46.94, 123.31 and 9.30, respectively. The influence of three factors on the adsorbed performance of ECSBC was: heating temperature (*X*_2_) > heating rate (*X*_3_) > heating time (*X*_1_)^46,47^, and the influence of interaction term is: *X*_1_ and *X*_2_ (heating time and heating temperature) > *X*_2_ and *X*_3_ (heating temperature and heating rate) > *X*_1_ and *X*_3_ (heating time and heating rate).

6 $$\begin{array}{c}{Y}_{R \% }=84.42+2.40{X}_{1}-3.88{X}_{2}+1.07{X}_{3}-2.20{X}_{1}{X}_{2}+1.47{X}_{1}{X}_{3}-2.06{X}_{2}{X}_{3}\\ \,\,\,-2.33{X}_{1}^{2}-21.78{X}_{2}^{2}-9.63{X}_{3}^{2}+5.71{X}_{1}^{2}{X}_{2}+0.95{X}_{1}^{2}{X}_{3}-2.41{X}_{1}{X}_{2}^{2}\end{array}$$

7 $$\begin{array}{c}{Y}_{q}=21.10+0.60{X}_{1}-0.97{X}_{2}+0.27{X}_{3}-0.55{X}_{1}{X}_{2}+0.37{X}_{1}{X}_{3}-0.51{X}_{2}{X}_{3}-0.58{X}_{1}^{2}\\ \,\,\,-5.44{X}_{2}^{3}-2.41{X}_{3}^{2}+1.43{X}_{1}^{2}{X}_{2}+0.24{X}_{1}^{2}{X}_{3}-0.60{X}_{1}{X}_{2}^{2}\end{array}$$

**Table 4:** The cubic regression model of ANOVA for adsorption capacity of Cd^2+^ by ECSBC.

Sources	Sum of Squares	df	Mean Square	F-value	p-value Prob. >F
(a) Remvoval rate Model	2720.23	12	226.69	463.39	<0.0001
*X*_1_	22.96	1	22.96	46.94	0.0024
*X*_2_	60.32	1	60.32	123.31	0.0004
*X*_3_	4.55	1	4.55	9.30	0.0380
*X*_1_*X*_2_	19.36	1	19.36	39.58	0.0033
*X*_1_*X*_3_	8.65	1	8.65	17.69	0.0136
*X*_2_*X*_3_	16.95	1	16.95	34.64	0.0042
*X*_1_^2^	22.90	1	22.90	46.82	0.0024
*X*_2_^2^	1996.99	1	1996.99	4082.26	<0.0001
*X*_3_^2^	390.65	1	390.65	798.58	<0.0001
*X*_1_*X*_2_*X*_3_	0.000	0	—	—	—
*X*_1_^2^*X*_2_	65.17	1	65.17	133.22	0.0003
*X*_1_^2^*X*_3_	1.79	1	1.79	3.66	0.1284
*X*_1_*X*_2_^2^	11.64	1	11.64	23.80	0.0082
*X*_1_*X*_3_^2^	0.000	0	—	—	—
*X*_2_^2^*X*_3_	0.000	0	—	—	—
*X*_2_*X*_3_^2^	0.000	0	—	—	—
*X*_1_^3^	0.000	0	—	—	—
*X*_2_^3^	0.000	0	—	—	—
*X*_3_^3^	0.000	0	—	—	—
Pure error	1.96	4	0.49	—	—
Cor total	2722.19	16	—	—	—
(b) Adsorption capactiy Model	170.01	12	14.17	463.39	<0.0001
*X*_1_	1.44	1	1.44	46.94	0.0024
*X*_2_	3.77	1	3.77	123.31	0.0004
*X*_3_	0.28	1	0.28	9.30	0.0380
*X*_1_*X*_2_	1.21	1	1.21	39.58	0.0033
*X*_1_*X*_3_	0.54	1	0.54	17.69	0.0136
*X*_2_*X*_3_	1.06	1	1.06	34.64	0.0042
*X*_1_^2^	1.43	1	1.43	46.82	0.0024
*X*_2_^2^	124.81	1	124.81	4082.26	<0.0001
*X*_3_^2^	24.42	1	24.42	798.58	<0.0001
*X*_1_*X*_2_*X*_3_	0.000	0	—	—	—
*X*_1_^2^*X*_2_	4.07	1	4.07	133.22	0.0003
*X*_1_^2^*X*_3_	0.11	1	0.11	3.66	0.1284
*X*_1_*X*_2_^2^	0.73	1	0.73	23.80	0.0082
*X*_1_*X*_3_^2^	0.000	0	—	—	—
*X*_2_^2^*X*_3_	0.000	0	—	—	—
*X*_2_*X*_3_^2^	0.000	0	—	—	—
*X*_1_^3^	0.000	0	—	—	—
*X*_2_^3^	0.000	0	—	—	—
*X*_3_^3^	0.000	0	—	—	—
Pure error	0.12	4	0.031	—	—
Cor total	170.14	16	—	—	—

### Interaction effects of factors and response surface

**Figure 4:**
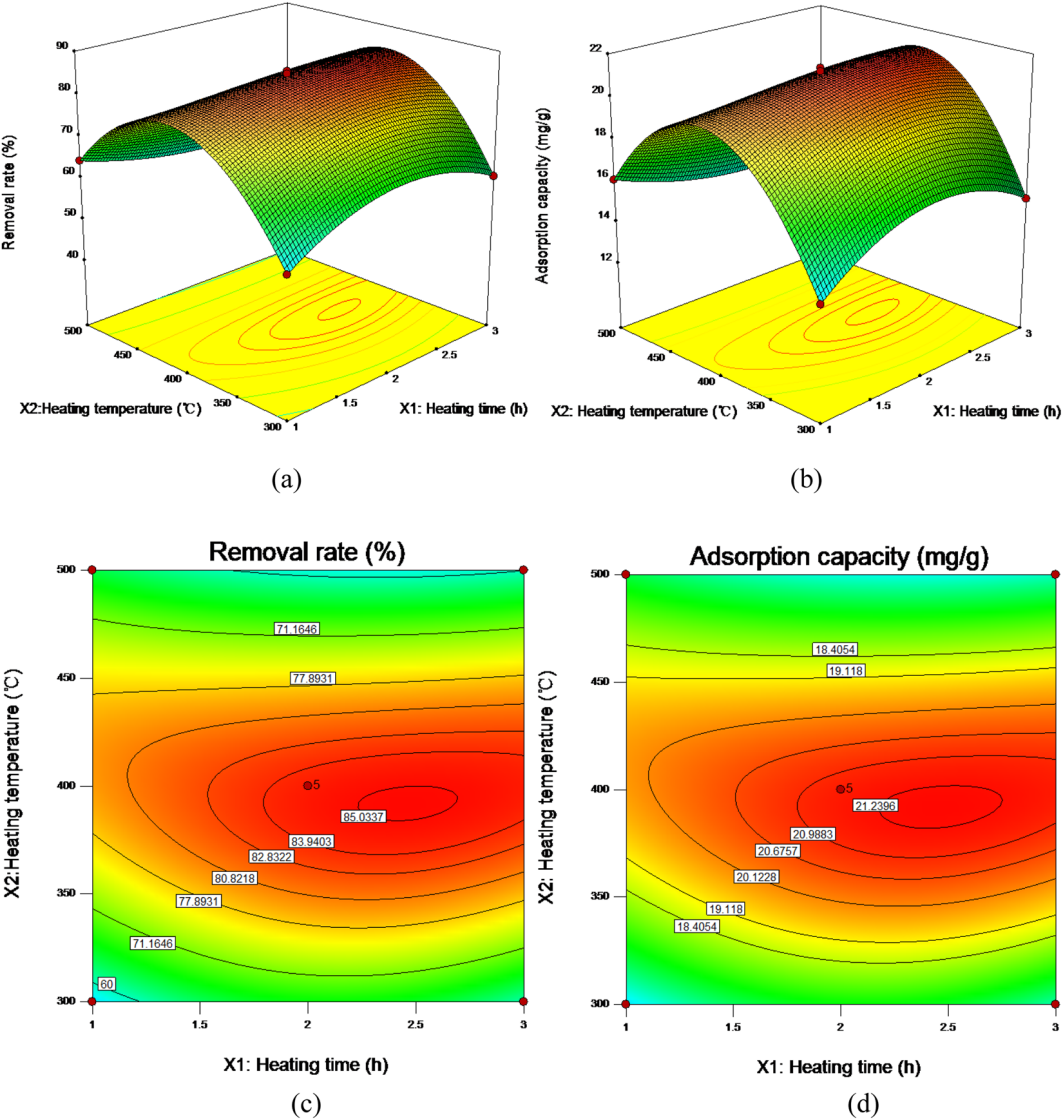
Three-dimensional (3D) response surface plot and contour diagram of the interaction of heating time and heating temperature for removal rate: (**a**) three-dimensional (3D) response surface plot for removal rate; (**b**) three-dimensional (3D) response surface plot for adsorption capacity; (**c**) contour diagram for removal rate; (**d**) contour diagram for adsorption capacity.

**Figure 5:**
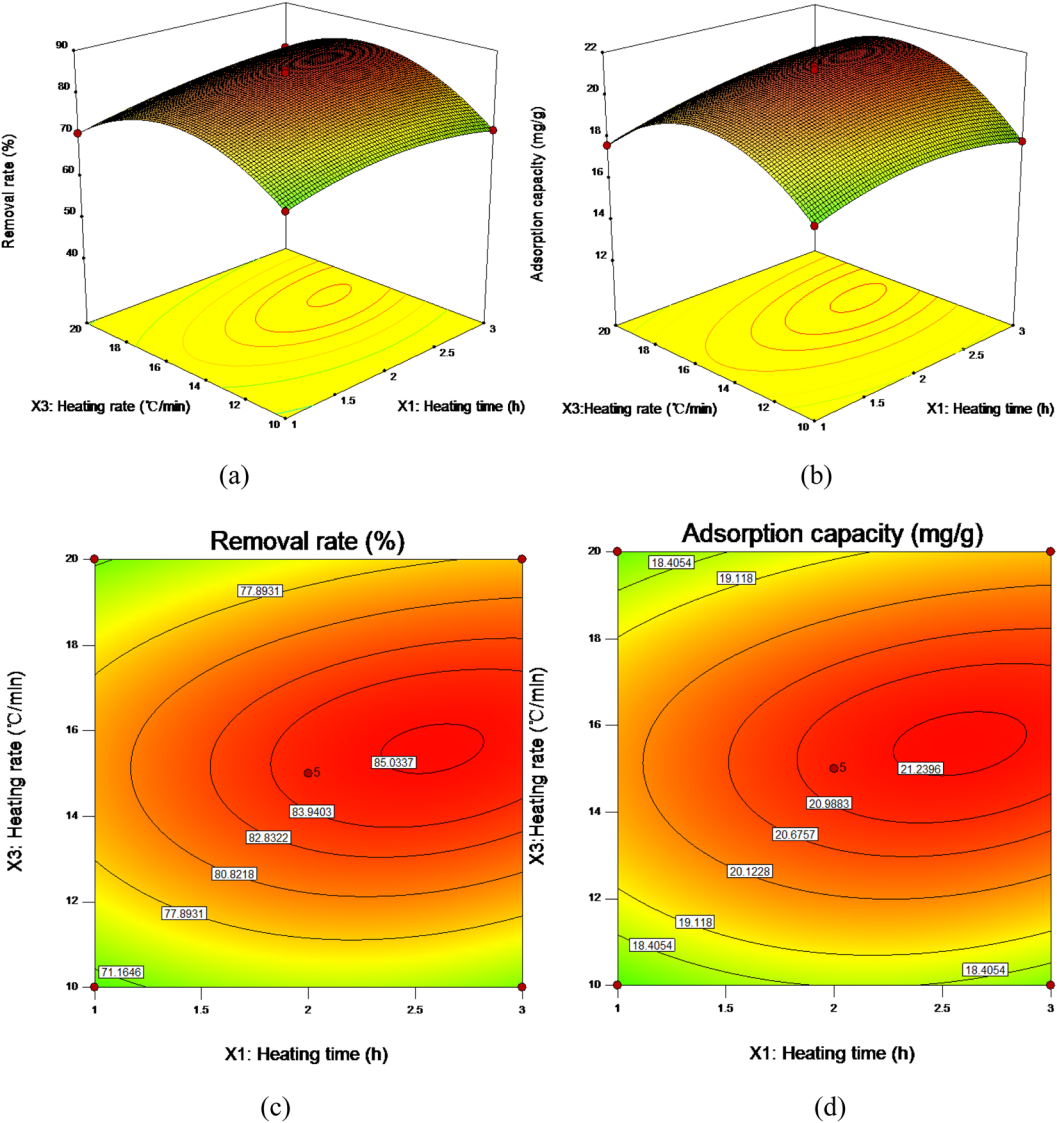
Three-dimensional (3D) response surface plot and contour diagram of the interaction of heating time and heating rate: (**a**) three-dimensional (3D) response surface plot for removal rate; (**b**) three-dimensional (3D) response surface plot for adsorption capacity; (**c**) contour diagram for removal rate; (**d**) contour diagram for adsorption capacity.

**Figure 6:**
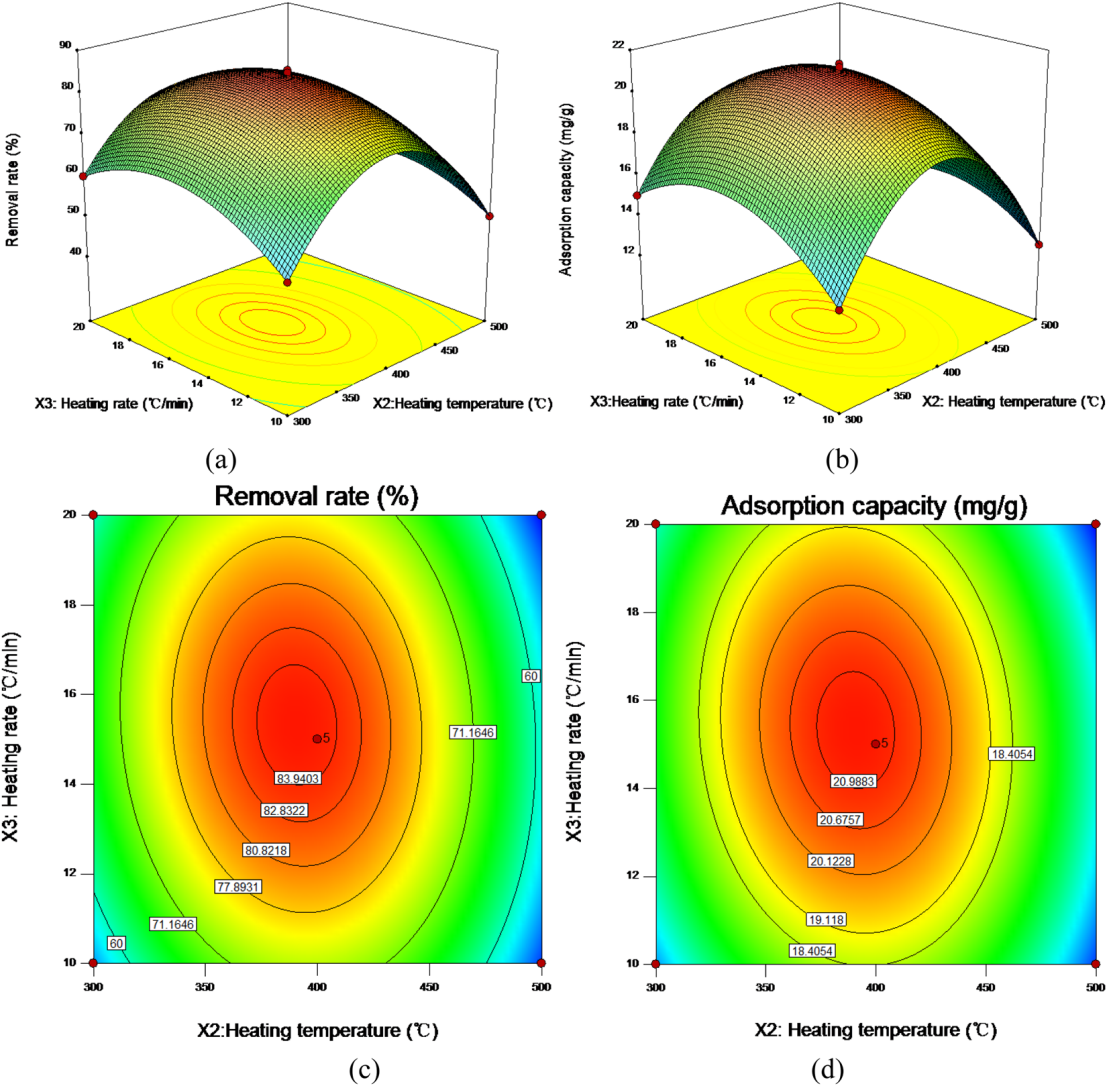
Three-dimensional (3D) response surface plot and contour diagram of the interaction of heating temperature and heating rate: (**a**) three-dimensional (3D) response surface plot for removal rate; (**b**) three-dimensional (3D) response surface plot for adsorption capacity; (**c**) contour diagram for removal rate; (**d**) contour diagram for adsorption capacity.

From the cubic multiple regression models, the response surface three-dimensional diagram and contour diagram of the interaction of pyrolysis time (*X*_1_), pyrolysis temperature (*X*_2_) and heating rate (*X*_3_) on the adsorption capacity and removal rate of Cd^2+^adsorbed by ECSBC can be obtained. The results were shown in Figs. 4(a–d)–6(a–d).

It can be seen from Fig. 4(a–d) that the response surface of *X*_1_ and *X*_2_ for removal rate and adsorption capacity has a steep slope, and the contour diagram presents an elliptical shape, indicating significant interaction between the two factors^48^. Therefore, *X*_1_ and *X*_2_ play an important role in preparing ECSBC for adsorbing Cd^2+^, and their changes have a common effect on Cd^2+^ adsorbed by ECSBC. Therefore, the horizontal combination of the two factors should be investigated when seeking the optimal preparation conditions. When *X*_2_ is fixed, the removal rate and adsorption capacity change little with the increase of *X*_1_, and when *X*_1_ fixed, the removal rate and adsorption capacity first increased and then decreased with *X*_2_ increased, and the trend of increase and decrease were obviously. The higher removal rate and adsorption capacity values appeared around *X*_1_ of 2.5 h and *X*_2_ was about 400 °C.

According to Fig. 5(a,d), the interaction between *X*_1_ and *X*_3_ on removal rate and adsorption capacity is lower than that between *X*_1_ and *X*_2_. The contour line also presented elliptic shape, indicating that the interaction was still significant, but less significant than the interaction between *X*_1_ and *X*_2_. Figure 6(a,d) showed the effects of the interaction between *X*_2_ and *X*_3_ on removal rate and adsorption capacity. When the *X*_3_ is constant, the removal rate and adsorption capacity of Cd^2+^ by ECSBC increased first and then decreased with the increase of *X*_2_. However, when the *X*_2_ was constant, the removal rate and adsorption capacity along with the change of *X*_3_ did not change as significantly as the *X*_2_. The 3D diagram of the interaction between *X*_2_ and *X*_3_ presents a certain slope, and the contour diagram also presents an elliptic shape. From *F* value and *P* value in Tables 3 and 4, it can be concluded that the order of influence of interaction term on the adsorption properties of Cd^2+^ by ECSBC is: *X*_1_ and *X*_2_ > *X*_2_ and *X*_3_ > *X*_1_ and *X*_3_.

### Verification of the model

**Figure 7:**
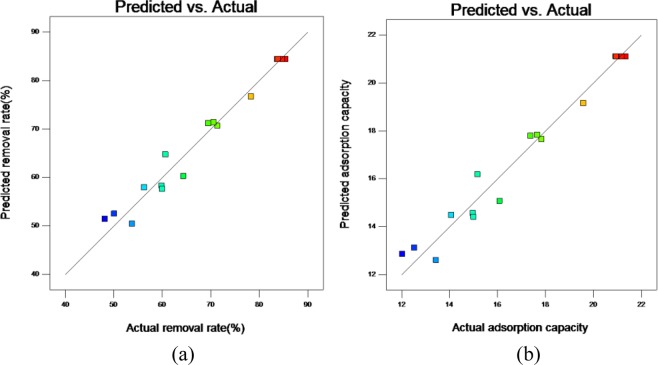
Relationship between predicated and experimental data for response: (**a**) removal rate (*R%*); (**b**) adsorption capacity (*q*_*t*_).

Through Design-expert10.0 software, three factors affecting the adsorption performance ECSBC adsorbed Cd^2+^ were obtained: The optimal combination of pyrolysis time, pyrolysis temperature and heating rate was 2.42385 h, 392.997 °C and 15.559 °C/min, respectively. According to analysis of RSM fitting model, the predicated removal rate and adsorption capacity were 85.2724% and 21.168 mg/g, respectively. In order to verify the predicted values, experiments were carried out under the optimum conditions: the heating time at 145 min, the heating temperature at 393 °C and the heating rate of 15.56 °C/min. The results of verification tests were shown in Table 5, and the relationship between the predicated removal rate and adsorption capacity and the actual removal rate and adsorption capacity were displayed in Fig. 7(a,b). The actual removal rate and adsorption capacity were 80.70% and 20.175 mg/g, respectively, the deviation from the predicted value were 5.36% and 4.69%, and the error between the predicted value and the actual experimental value is small. Therefore, the RSM models were able to predict the removal rate and adsorption capacity of Cd^2+^ by OECSBC.

**Table 5:** The results of verification tests by OECSBC.

Response values (*Y*)	Predicted value	Experimental value 1	Experimental value 2	Experimental value 3	The average of experimental value
*R*(%)	85.2724	83.0	79.80	79.30	80.70
*Q*(mg/g)	21.168	20.75	19.95	19.825	20.175

### Adsorption kinetics and adsorption isotherms

To study the Cd^2+^ adsorption rate and mechanism of adsorption process by OECSBC, 1.0 g OECSBC was carried into 1000 mL corked conical flask and added 500 mL Cd^2+^ solution with initial concentration of 50 mg/L (other experimental conditions were: temperature at 298 K, rotate speed at 150 rpm/min). Samples were taken at 30, 60, 90, 120, 150, 180, 210, 240, 270, and 300 min, respectively, and determined the concentration of Cd^2+^ in filtrate. The pseudo first and second order models were used to analyze the adsorption kinetic data, two models listed followed:

Pseudo-first model^49^:

$${q}_{t}={q}_{e}(1-exp(-{k}_{1}t))$$

Pseudo-second model^49^:

$${q}_{t}=\frac{{q}_{e}^{2}{k}_{2}t}{1+{q}_{e}{k}_{2}t}$$

Where *q*_*e*_ and *q*_*t*_ (mg/g) are adsorption capacity of Cd^2+^ at equilibrium and *t* time (h), respectively, *k*_1_ (min^−1^)and *k*_2_ (g/mg·min) are the constants for pseudo-first and pseudo-second order models, respectively.

**Figure 8:**
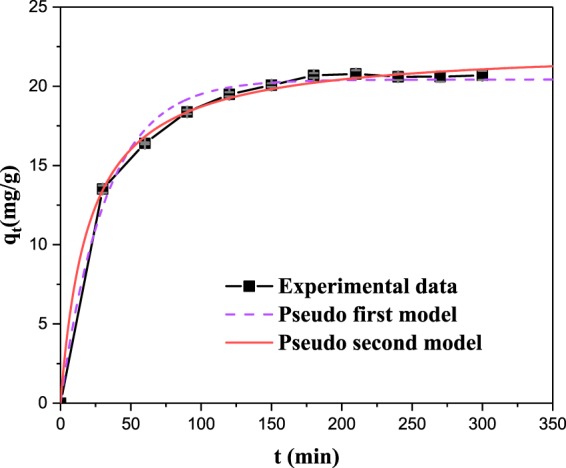
Adsorption kinetics of Cd^2+^ on the OECSBC.

The fitting results of two models on the experimental data are shown in Fig. 8 and Table 6. According to Fig. 8 and Table 6, the adsorption of Cd^2+^ by was in good fitted with the pseudo-first and pseudo-second order kinetics models, where the fitting coefficient were better by second order model (*R*^2^ = 0.98161) than first order model (*R*^2^ = 0.93016). The result showed that adsorption of Cd^2+^ by OECSBC was dominated by chemical adsorption^50^.

**Table 6:** Kinetic parameters for the adsorption of Cd^2+^ on the OECSBC

Pseudo first-order adsorption kinetic			Pseudo second-order adsorption kinetic		
*q*_*e*_ (mg/g)	*K*_*t*_ (min^−1^)	*R*^2^	*q*_*e*_ (mg/g)	*K*_2_ (g/mg·min)	*R*^2^
20.42019	0.03106	0.93016	22.49034	0.0022	0.98161

In this study, the experiments of removal of Cd^2+^ on the OECSBC were studied at different initial concentrations (1–1000 mg/L) at conditions about the adsorbent dosage of 2.0 g/L, pH 6.0, and reaction time at 300 min (other experimental conditions were: temperature at 298 K, rotate speed at 150 rpm/min). The data of the equilibrium was analyzed based on two isotherms models of Langmuir nonlinear model and Freundlich nonlinear model, two mathematic models were listed below:

Langmuir isotherm nonlinear model^51^:

$${q}_{e}=\frac{{K}_{a}{Q}_{m}{C}_{e}}{1+{K}_{a}{C}_{e}}$$

Freundlich isotherm nonlinear model^51^:

$${Q}_{e}={K}_{F}{C}_{e}^{\frac{1}{n}}$$

where *C*_*e*_ (mg/L) is solution concentration of sorbent at equilibrium, *K*_*a*_ is the nonlinear Langmuir constant, *K*_*F*_ is nonlinear Freundlich constant, *n* (Freundlich exponent) is an indicator of intensity change during adsorption process and also an index of deviation from linearity of adsorption. *Q*_*m*_ denote the nonlinear Langmuir maximum adsorption capacity (mg/g). *Q*_*e*_ is Cd^2+^ adsorption capacity at equilibrium (mg/g).

**Figure 9:**
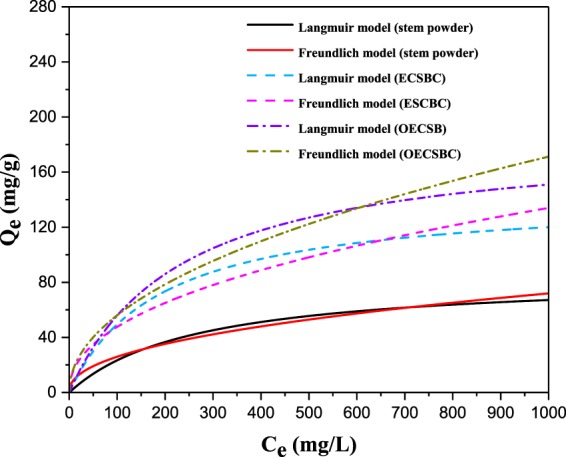
Adsorption isotherms of raw stem, ECSBC and OECSBC.

The results of two models fitting are listed in Fig. 9 and Table 7. According to values of *R*^2^, the Langmuir isotherm showed the best fitted values for OECSBC (*R*^2^ = 0.9840) and ECSBC (*R*^2^ = 0.9675), and this model indicated that the adsorption process occurs at a completely homogeneous adsorption sites, and each molecule possessing constants adsorption sites^52^. While the Frenudlich isotherm fitted well for the raw stem powder (*R*^2^ = 0.9747). As shown in Table 6, the maximum adsorption capacity of 84.79 mg/g, 142.59 mg/g and 186.18 mg/g for raw stem, ECSBC and OECSBC, respectively. The results showed that the OECSBC has strong adsorption capacity for Cd^2+^, and it is similar to the previous study of heavy metal adsorption in *Eichhornia crassipes*^53^.

**Table 7:** Parameters of nonlinear models of Langmuir and Freundlich for raw stem, ECSBC and OECSBC.

Models		OECSBC	ECSBC	Raw stem
Nonlinear-Langmuir	*K*_*a*_	0.004288	0.005314	0.003805
	*Q*_*max*_ (mg/g)	186.18	142.59	84.79
	*R*^2^	0.9840	0.9675	0.9019
Nonlinear-Freundlich	*K*_*F*_	6.0188	6.0223	3.9989
	*1/n*	0.4847	0.4491	0.4400
	*R*^2^	0.9761	0.9318	0.9747

### Characterization of OECSBC and adsorption mechanisms of Cd^2+^ by OECSBC

To study the adsorption mechanisms of OECSBC on Cd^2+^, the Zeta and pH of OECSBC were determined and the SEM-EDX, FTIR and XRD were used to analyze OESCBC before and after adsorption. It is concluded that the adsorption mechanisms of OECSBC on Cd^2+^ mainly includes precipitation, electrostatic adsorption, surface physical adsorption, ion exchange and complexation of functional groups.

#### Precipitation and electrostatic adsorption

The pH of OECSBC was 9.33, and the pH of Cd^2+^ for precipitation is 9.0^54^, so the precipitation is one of the adsorption mechanisms of Cd^2+^ by OECSBC. The Zeta potential of OECSBC was about 2.50, which indicates that when the pH of solution >2.50, OECSBC has a negative charge on its surface, and a strong electrostatic adsorption will occur between OECSBC and Cd^2+^.

#### Surface physical adsorption

**Figure 10:**
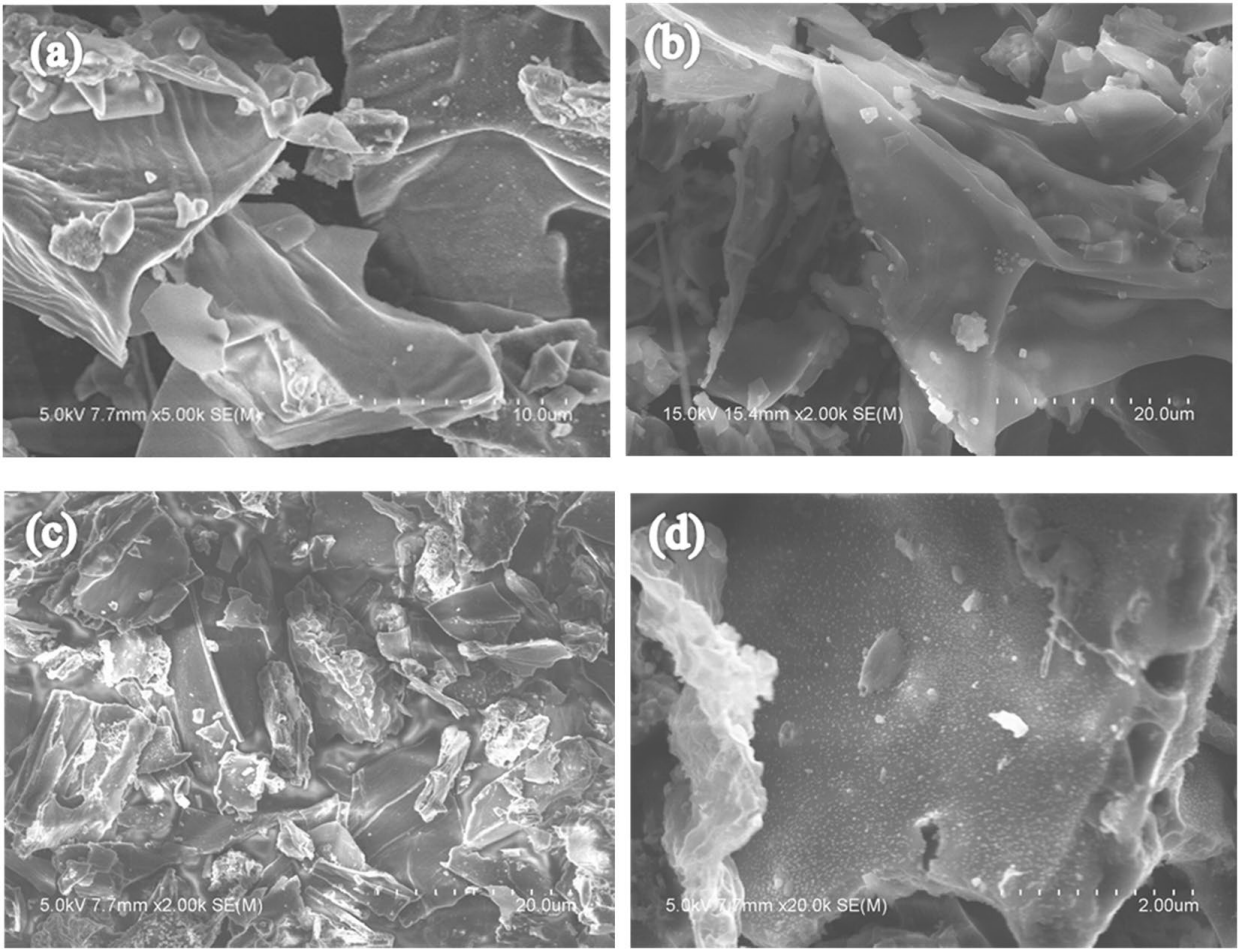
SEM images of OECSBC and OECSBC + Cd: (**a**,**b**) OECSBC; (**c**,**d**) OECSBC + Cd.

The scanning electron microscope (SEM) images of OECSBC and OECSBC + Cd (the OECSBC after adsorption of Cd^2+^) are shown in Fig. 10(a–d). The SEM images of the OECSBC particles showed smoother surface, and displayed a lots of fragments, and these stacked fragments allow heavy metal ions to enter the pores of the biochar. But the SEM images of OECSBC + Cd displayed a lots of obvious crystal particles on the surface of the biochar, and the EDX analysis showed that these particles contain Cd element.

According to the BET and pore size distribution analysis, the average pore diameter of OECSBC and raw stem are 48.637 nm and 11.265 nm, respectively, which are belong to mesoporous materials and suitable for adsorption materials^55^. The surface area of OECSBC (4.436 m^2^/g) is 20.54 times as much as that of raw stem (0.216 m^2^/g), and it can be provide a bigger contact area to adsorb more Cd^2+^, and the BJH adsorption pores volume of OECSBC (0.009 cm^3^/g) is 4.5 times greater than that of the raw stem (0.002 cm^3^/g). In summary, surface physical adsorption is a mechanism for OECSBC to adsorb Cd^2+^.

#### Ion exchange

The EDX spectrums of OECSBC and OECSBC + Cd are showed in Table 8. Through the EDX elemental analysis, the content of K and Ca were decreased after adsorption. So the results showed that the K, and Ca play an ion exchange in adsorption process^56^.

**Table 8:** EDX elemental analysis of OECSBC and OECSBC + Cd.

Element (Wt%)	OECSBC	OECSBC + Cd
C	29.40	76.97
O	6.01	19.42
K	28.66	0.51
Mg	0.22	0.44
Ca	1.43	1.01
P	0.40	0.67
Cl	33.88	—
Cd	—	0.97
Totals	100.00	100.00

**Figure 11:**
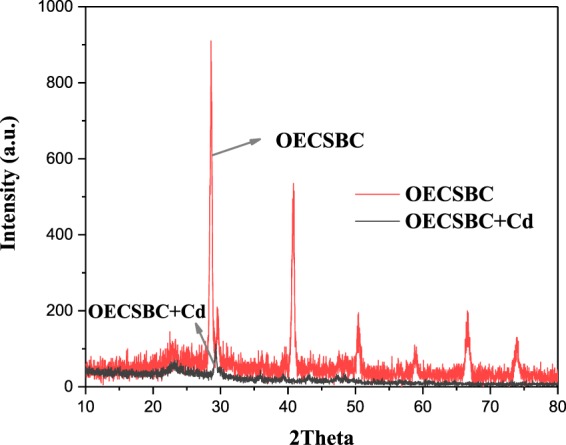
XRD patterns of OECSBC and OECSBC + Cd.

The XRD patterns of OECSBC and OECSBC + Cd are displayed in Fig. 11. It can be seen from Fig. 11 that OECSBC contains a large amount of KCl and a certain amount of CaCO_3_^57^. However, in the pattern of OECBSC + Cd, these peaks were changed or disappeared. It is shown that after adsorption of Cd^2+^, K^+^ and Ca^2+^ had ion exchange reaction with Cd^2+^.

#### Complexation of functional groups

**Figure 12:**
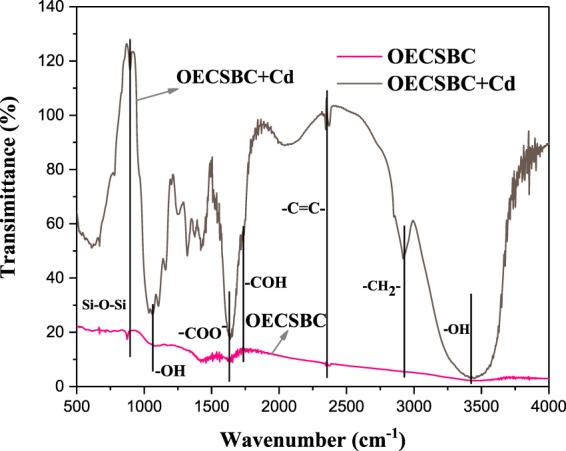
FTIR spectra of OECSBC and OECSBC + Cd.

The surface functional groups is the main chemical factor affecting adsorbents adsorption of heavy metals. In order to indentify the functional groups on the surface of three adsorbents, the Fourier Transform Infrared Spectroscopy (FTIR) was used, and the spectra of OECSBC and OECSBC + Cd were depicted in Fig. 12. From Fig. 12, the bands around 3500–3200 cm^−1^, 2800–2900 cm^−1^, 2400–2500 cm^−1^, 1656 cm^−1^, 1100–1200 cm^−1^ and 800 cm^−1^ were belonged to the strecthing vibrations of the –OH, –CH_2_–, –C = C–, –CHO–, –COO–, –OH and Si–O–Si functional groups, while in spectra of OECSBC + Cd, the width and position of these peaks changed. According to the above analysis, adsorption of Cd^2+^ by OECSBC is related to –OH, –CH_2_–, –C = C–, –CHO–, –COO–, –OH and Si–O–Si functional groups, which is consistent with studies of Gao *et al*. and Qiu *et al*.^58,59^.

**Figure 13:**
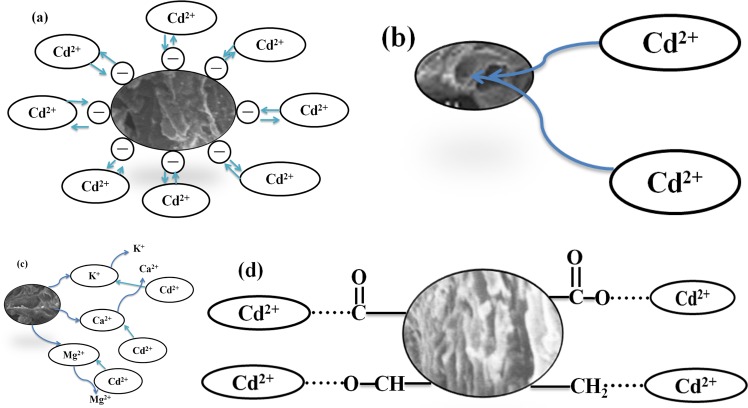
OECSBC adsorption mechanisms for Cd^2+^: (**a**) electrostatic adsorption; (**b**) ion exchange; (**c**) complexation of functional groups; (**d**) Surface physical adsorption.

In conclusion, adsorption of Cd^2+^ by OECSBC is not only related to surface structure, but also related to surface functional groups and mineral components. Adsorption mechanisms of OECSBC on Cd^2+^ are shown in Fig. 13. According to the pH, Zeta potential and other characterization methods, the adsorption mechanisms of OECSBC on Cd^2+^ are mainly include: (a) precipitation and electrostatic adsorption; (b) ion exchange; (c) complexation of functional groups; (d) surface physical adsorption.

### Comparison with other adsorbents for adsorption of Cd^2+^

The adsorption capacity of biochar materials to Cd^2+^ is different with different raw materials and preparation methods. The Cd^2+^ adsorption capacities of adsorbents have been published in previous studies are summarized in Table 9. By comparing the adsorption performance of different biochar to Cd^2+^ in Table 9, it can be concluded that the theoretical adsorption capacity of Cd^2+^ by OECSBC is relatively larger than others except the *Canna indica* biochar (500 °C)^60^. However, *Eichhornia crassipes* belongs to aquatic plant, and its adsorption capacity to heavy metals is different under different water quality conditions, the biochar prepared from *Eichhornia crassipes* which grow up with high content of N and P may have stronger adsorption ability to heavy metals than it with heavy metal pollution. Therefore, for OECSBC adsorption of heavy metals, the source of *Eichhornia crassipes* should be strictly controlled. In this study, *Eichhornia crassipes* was taken from the pond in the campus, which was mainly sewage discharged from the domestic sewage and the canteen, and the content of N and P was high, while heavy metal ions such as Cd^2+^ were not detected.

**Table 9:** Comparison of Cd^2+^ adsorption capacity (*Q*_*m*_) with other reported adsorbents.

Adsorbents	Cd^2+^ adsorption capacity (mg/g)	References
*Canna indica* biochar (300 °C)	63.32	^60^
*Canna indica* biochar (400 °C)	105.78	
*Canna indica* biochar (500 °C)	188.79	
*Canna indica* biochar (600 °C)	140.01	
*Ipomoea fistulosa* biochar (350 °C)	55.5	^61^
*Ipomoea fistulosa* biochar (400 °C)	71.43	
*Ipomoea fistulosa* biochar (500 °C)	62.5	
*Ipomoea fistulosa* biochar (550 °C)	41.67	
Activated biochar (ABC)	72.43	
Raw attapulgite (APT)	10.38	^62^
MgO modified APT (MAP/APT)	121.14	
Rice straw biochar (400 °C)	37.24	^63^
Rice straw biochar (700 °C)	65.40	
Palm oil mill sludge biochar	46.2	^64^
Staw stem	84.79	This study
ECSBC	142,59	This study
OECSBC	186.18	This study

From the Table 9, the OECSBC had comparatively much better adsorptive capacity to removal Cd^2+^ than other adsorbents, which suggested that the RSM can optimize the preparation conditions of ECSBC, and the OECSBC has highest adsorption capacity of Cd^2+^ among these adsorbents.

## Conclusions

Heating time, heating temperature and heating rate were considered as main factors on adsorption properties of *Eichhornia crassipes* stem biochar (ECSBC). The response surface methodology (RSM) using the Box-Behnken design (BBD) was applied to optimization of the preparation factors to maximize responses (adsorption capacity and removal rate for Cd^2+^). RSM displayed the heating temperature showed the strongest effect on adsorptive property of ECSBC, and the optimal preparation conditions were heating time of 2.42 h, heating temperature of 393 °C, and heating rate of 15.56 °C/min. Under the optimum conditions, the actual removal rate and adsorption capacity were 80.70% and 20.175 mg/g, respectively, which were in accordance with the predicted values 85.2724% and 21.168 mg/g, respectively, and the maximum adsorption capacity of Cd^2+^ could reach 186.18 mg/g. The pH, Zeta, BET and pore size distribution, SEM-EDX, FTIR and XRD analysis before and after Cd^2+^ adsorption by OECSBC showed that the mechanisms of OECSBC are mainly through ion exchange reaction between Cd^2+^ and soluble metal ions K^+^, Ca^2+^ and Mg^2+^ on the surface of OECSBC, precipitation reaction with –OH, $P{O}_{4}^{3-}$and $C{O}_{3}^{2-}$, complexation reaction with surface –C = O–, –COO–, –CHO and –CH_2_– functional groups and physical adsorption, so as to achieve Cd^2+^ removal. The results showed the RSM could optimize the preparation of conditions of the ECSBC, and the OECSBC has a strong adsorption capacity of Cd^2+^, so it can be as a potentially low-cost biosorbent for removal Cd^2+^ from aqueous solutions.
